# Influence of Music on Closed Motor Skills: A Controlled Study with Novice Female Dart-Throwers

**DOI:** 10.3390/ijerph17114146

**Published:** 2020-06-10

**Authors:** Félix Arbinaga, Nehemías Romero-Pérez, Lidia Torres-Rosado, Eduardo J. Fernández-Ozcorta, María Isabel Mendoza-Sierra

**Affiliations:** 1 Department of Clinical and Experimental Psychology, Faculty of Education, Psychology and Sports Science, University of Huelva, 21007 Huelva, Spain; lidia.torres.rosado@hotmail.com; 2 Department of Social, Evolutionary and Educational Psychology, Faculty of Education, Psychology and Sports Science, University of Huelva, 21007 Huelva, Spain; nemimantroni@yahoo.es (N.R.-P.); imendoza@dpsi.uhu.es (M.I.M.-S.); 3 Department of Physical Activity and Sports, Center for University Studies Cardenal Spínola CEU, University of Seville Attached Centre, 41930 Sevilla, Spain; ejfernandez@ceuandalucia.es

**Keywords:** tempo, attention, performance, music, synchronization, motor tasks, closed skills, throwers

## Abstract

The influence of music heard at different tempos is analyzed during the execution of a dart-throwing task. The sample consisted of 56 female university students (Mean age = 23.38, SD = 6.773). The participants were randomly assigned to GC (group control without music; *n* = 18), GS (group with slow-paced music at a tempo of 60 BPM; *n* = 19) and GF (group with fast-paced music at a tempo of 105 BPM; *n* = 19). All participants performed a dart-throwing task in two phases. Analysis of the scores obtained during Phase 1 and Phase 2 of dart throwing (examining both between-group differences and within-group differences, i.e., changes in scores from Phase 1 to Phase 2 using a mixed factorial ANOVA) revealed no differences in dart-throwing scores. There were, however, differences in execution time, where the participants in GS needed more time to complete the task than those in GF (*F_(2,55)_* = 4.426, *p* = 0.017) with a large effect size (*ŋ^2^_p_* = 0.143), although neither of these groups differed from GC. The results are discussed in terms of the role of music in precision tasks and the synchronization of the task with the pace of the music.

## 1. Introduction

Music has been shown to significantly affect performance and task execution [1]. The available evidence on this topic suggests that music can consistently and measurably promote physical activity (as measured by psychophysiological parameters and exercise-related performance) and encourage rhythmic movement [2].

A number of studies have compared work-related performance in the presence and absence of music. For instance, Burleson, Center, and Reeves [3] found that background music facilitated performance on a color-coded sorting task in psychotic children, whilst in a study of adults with intellectual disabilities, Groeneweg, Stan, Celser, MacBeth, and Vrbancic [4] reported that listening to music had a positive impact on productivity during a grocery coupon sorting task, with greater task accuracy and a reduction in non-work related behaviors. The features of the music, including the time at which it is played, the tempo, and the way in which it is presented (contingent or non-contingent with respect to the task) should be carefully selected to suit the requirements of the task and the characteristics of the individuals involved [5,6].

Rhythm is a basic component of musical composition [1]. In musical terms, rhythm is defined as the temporal organization of the musical material classified by the onset and duration of stimuli, and the intervals between those onsets [7], whilst tempo is defined as the speed of a composition’s rhythm, and is measured according to beats per minute (BPM), which is generally accepted to be an adequate measure of musical speed [8].

Many investigators have studied the concept of entrainment or the ability to synchronize motor behavior with auditory sequences [8] using simple isochronous and/or tempo-modulated tone sequences as stimuli [2,9]. Motor entrainment is explicitly driven by active interventions, but not by those that emphasize passive listening without motor coordination. Motor entrainment is attributed to the differential modulation of the sympathetic activity of the autonomic nervous system (ANS) [10]. Even though the physiological mechanisms are unclear, the synchronization of movement speed with the tempo of music improves performance on gross motor tasks [10]. For instance, one study evaluated the impact of fast/slow tempo music on 500 m rowing sprint performance [11]. Twenty-two rowers performed 500 m sprints on three occasions: without music, with slow music (allegretto), and with fast tempo music (allegro con brio). Strokes per minute (SPM), time to completion (TTC), and rated perceived exertion (RPE) were recorded. Although there were no differences in RPE between the rowing conditions, TTC was shortest in the fast tempo music condition. Further, a shorter TTC was observed in the slow music condition in comparison with the control condition, indicating that slow music also enhanced performance. The strongest effects emerged, however, when inspecting SPM, which was found to be significantly higher during rowing to fast music in comparison with rowing to slow music or no music. These results suggest that fast music could act as an external “psyching-up” stimulus for brief and strenuous muscle work.

In the sporting context, the role of music in relatively sedate target sports (e.g., bowls, darts, archery, boules, and shooting sports, among others) is essentially to mentally prepare competitors. In terms of the mental focus needed, individual sports that require fine motor skills (e.g., billiards, archery, darts, and target shooting) are very different from those that require gross motor skills, such as powerlifting and shot putting [5]. Although the research concerned with the effects of background music has yielded mixed results, there is evidence to suggest that this (background music that is either contingent or noncontingent with respect to the task) could be an effective strategy for increasing task engagement and performance [12]. In the specific field of sport, when studying the relationship between musical rhythm and performance [5], the results indicate that exercise is more efficient when performed synchronously with music when the tempo is slightly slower than the rate of cyclical movement [13].

In sports where fine motor skills are required (e.g., dart throwing), some studies have evaluated the influence of music in the time periods preceding competitive activity. The objective of this pre-task music is to eliminate distractions and isolate the competitor from the environment, thereby producing an optimal competitive state [5]. However, whilst there is considerable inter-individual variation in terms of physiological and mental arousal, most players benefit from attaining a calm and focused state of mind (e.g., through visualization training). In this context, music can be effective in stimulating a person to respond to simple and repetitive tasks as opposed to those of a more complex nature [12].

Studies analyzing the use of pre-task music have found positive effects on performance. For example, Kuan, Morris, Kueh, and Terry [14] investigated the effects of playing relaxing and arousing music (during imagery training) on the scores obtained by 63 novice dart throwers. The participants were assigned to groups exposed to unfamiliar relaxing music (URM), unfamiliar arousing music (UAM), or no music (NM). The results revealed that dart-throwing scores were significantly higher in the URM than the UAM and NM groups, with no significant differences between the latter two groups. Whilst participants in all three conditions showed improved performance, the URM condition was associated with the highest gain in performance, the lowest physiological rates of arousal, and more adaptive activation profiles. Thus, listening to relaxing music during imagery could enhance performance on other tasks that require fine motor skills.

However, the presence of music during the performance of precision sports (e.g., billiards, bowling, darts, and archery, among others) could serve as a distracting stimulus [15] or generate physiological conditions incompatible with the task, since high-tempo music is usually associated with an increase in heart rate [16], which can hinder performance [5]. In this regard, it has been suggested that tempos slower than the physiological heart rate could increase parasympathetic activation and suppress sympathetic activation of the autonomic nervous system (ANS) [10], thereby increasing the likelihood of observing an improved performance.

To the best of our knowledge, no studies have analyzed the influence of music played through headphones during the performance of precision sports (e.g., billiards, bowling, darts, and archery). One reason for this could be the fact that the use of music has often been discouraged due to its possible distracting effects when players need to practice a fine and demanding motor skill [6]. Thus, it has also been reported that music (defined as sounds having harmony, melody, or rhythm) could be as distracting as noise (unwanted auditory signals that could be disturbing or facilitating) when it comes to human vigilance [15]. For instance, Brodsky [9] explored the effects of music on performance in a simulated driving and vehicle control task and found that high-tempo background music increased the number of driving errors and the frequency of virtual traffic offences.

Given the lack of studies concerning the influence of music when presented simultaneously during the execution of a task involving precision sports, the present study sought to address this gap in the literature by exploring the impact of music on performance when presented during a dart-throwing task. On the basis of the work reported by Karageorghis [5], we expected that the groups that threw the darts in the presence of music would show a poorer performance in terms of the scores obtained in comparison with a control group in which the task was executed without music. Further, we anticipated that the group presented with slow-tempo music would perform better on the task than the group presented with fast-tempo music. Finally, we hypothesized that the group presented with fast-tempo music would take less time to complete the task than both the control group without music and the group that performed the task whilst listening to slow-tempo music.

## 2. Materials and Methods

### 2.1. Design

The study was conducted using a randomized double-blind experimental design with three groups: a control group without music and two experimental groups that differed in terms of the tempo of the music presented (BPM).

### 2.2. Sample

**Figure 1:**
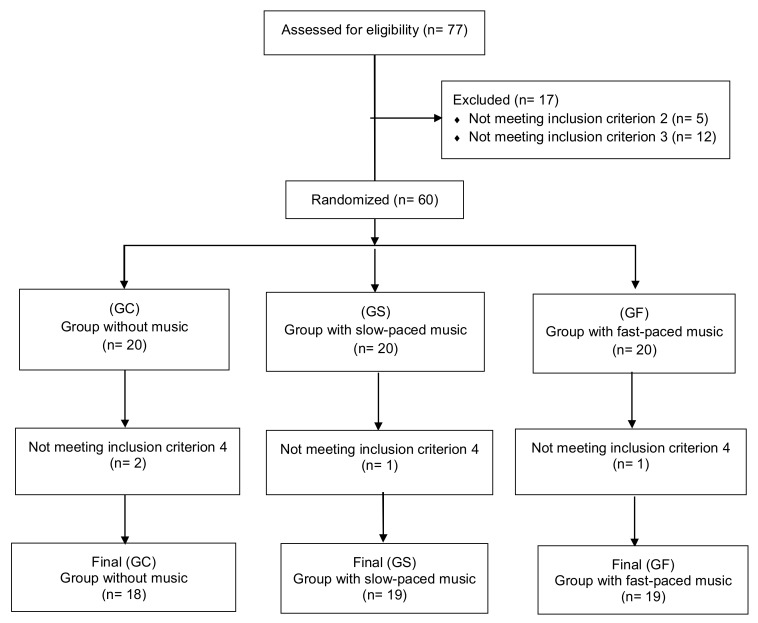
Flow diagram for the recruitment and group assignment of the study participants.

The study participants were 56 female volunteers with an average age of 23.38 years (SD = 6.773). Evidence suggests that it is deliberate practice and experience that often explains the observed differences in performance and skill levels between men and women during dart-throwing [17]. We therefore decided to include only women in our sample, given that one of our inclusion criteria was that the participants were novice dart players (see below). The participants were randomly assigned to the following groups: GC—control group without music (*n* = 18); GS—group slow, i.e., presented with slow-tempo music at 60 BPM (*n* = 19); and GF—group fast, presented with fast-tempo music at 105 BPM (*n* = 19). We recruited the participants by asking for volunteers in classes of the university. The inclusion criteria were: 1) being a female over 18 years of age; 2) having played darts less than once a month or never in the last 5 years; 3) obtaining a minimum score of 6 points on the scale administered to rate the piece of music (ranging from 0: “I like it, but very little” to 10: “I like it very much”); 4) completing the two phases of the task; and 5) providing written informed consent (see Figure 1).

### 2.3. Assessment Tools

An ad hoc interview was used to gather data related to year of birth, dart-playing experience (1. none; 2. very little/almost none; 3. quite a lot. 4. a lot) and were asked “how often have you played darts in the last 5 years” (1. never; 2. less than once a month; 3. between 2–5 times per month; 4. five or more times a month). Similarly, participants were asked, “do you have musical experience?” (Yes/No) “Do you have musical training?” (Yes/No), and “How many years of musical training do you have?” Potential participants were also asked to “indicate how much you like the music you have just heard”, on a scale from 0 to 10; with 0 indicating, “I like it, but very little” and 10 “I like it very much”.

The General Self-Efficacy Scale (GSS) [18] was used in its version adapted to the Spanish population [19]. In the present study, the scale had a Cronbach’s alpha coefficient of 0.759. The Life Orientation Test (LOT-R) [20], in its Spanish version [21], was used to assess the level of optimism in our participants. LOT-R consists of 10 items (four of which are control items). Of the six items, three are written in a positive way (optimism perspective) and three in a negative way (pessimism perspective). The items written in a negative way can be reversed in order to obtain a total score oriented towards the optimism dimension (Total Optimism). The Cronbach’s alpha value corresponding to LOT-R in the present study is 0.672. Finally, to assess state anxiety, we used the State-Trait Anxiety Inventory (STAI) [22] in its Spanish version [23]. In this study, this scale was shown to have a Cronbach’s alpha of 0.917.

### 2.4. Procedure

Given that optimism, self-efficacy, and anxiety have an impact on sporting performance [24,25,26], it was important to control for these effects on the current task. Therefore, these variables were evaluated prior to the onset of the experimental phases. 

Expectation manipulation: the objective of the study was hidden from the participants. In order to reduce the influence of demand characteristics during execution of the task [27], we proceeded to manipulate expectations about the real objective of the study during the phase of recruiting volunteers; they were informed that the objective of the investigation was to analyze performance on precision tasks under soundproofing conditions designed to control ambient noise. Further, at the end of the experiment (before revealing the real objective), in order to establish whether we had been able to conceal the objective from the participants, they were asked “what do you think was the objective of the activity you have just carried out?”

Musical composition: to ensure that the musical composition could influence performance [1], one of the inclusion criteria was that the musical style was suited to the taste of the participants. During the recruitment phase, the participants listened to a musical fragment at 80 BPM for four minutes. After this they were asked to rate the musical composition according to whether they liked it, on a scale ranging from 0 (“I like it, but very little”) to 10 (“I like it very much”). We excluded those participants who scored below 6 on this scale (Criterion 3). Further, to control for the effects of culture on task performance, as well as experience with musical compositions [1], we decided to develop an original musical piece (without lyrics), which was used in both the recruitment and execution phases.

After excluding candidates who did not meet the inclusion criteria, the remaining women were asked to complete the ad hoc interview and questionnaires (GSS, LOT-R). Subsequently, the participants were randomly assigned to three groups: the control without music group (GC); the slow-paced music group (GS), with music played at a tempo of 60 BPM; and the fast-paced music group (GF), with music played at a tempo of 105 BPM.

The choice of BPM (60 and 105 BPM) was determined by the fact that aiming and shooting sports require relatively low levels of physiological arousal and cognitive anxiety to achieve optimal performance. It has also been found that music is more relaxing at 60 BPM [28], whilst a high musical tempo is usually associated with an increase in heart rate [16]. Considering the latter, and to control for the influence of high musical tempo on the heart rate (in music, a high tempo is defined as one that is above 120 BPM [29]), we selected two tempos that are very close to the heart rate, which we labeled as slow tempo (60 BPM) and fast tempo (105 BPM). Moreover, since there appears to be a preference for music played at a tempo between 90 and 120 BPM [1], this allowed us to meet one of our criteria for ensuring that the music had an influence on performance, that is, that the music is pleasurable to the listener. The reason for all of this is that the required movements are fine, controlled, and must be extremely precise [5]. Further, to control for the influence of the loudness of the music, in the music groups the piece of music was played at 75 dB, since it is known that faster reaction times are obtained at this volume when compared with quieter music (60 dB) or loud music (greater than 80 dB) [15]. The decibel levels were measured using an iPhone 8 plus with the App: “Decibel X - dBA Sonometer”.

The music was recorded in a studio using a “MacBook Pro”. The program used for recording was “Logic Pro X” through the “Apogee Gio” audio interface. During the participant recruitment phase, the music was played in the classroom with a portable speaker (“MusicAngel”) from an iPhone 8 plus, all in MP3 format. In the laboratory, during both the warm-up phase and the throwing phase, wireless headphones (Sony) were used to play the tracks from the iPhone 8 plus (connected via Bluetooth). The musical characteristics of the piece are as follows: at the harmonic level, the tone of the song is in E major. Four different chord loops were used throughout the entire song, corresponding in Roman numerals to Grades I-V-Vim-IV.-Mi (E)-Si (B)-Do#m (C#m)-La (A). At the melodic level, the major scale of Mi (E).-Mi (E)-Fa# (F#)-Sol# (G#)-La (A)-Si (B)-Do# (C#)-Re# (D#) was used. Finally, at the rhythmic level, the base track was recorded at a tempo of 80 BPM. The base recording (80 BPM) was adjusted to the 60 BPM and 105 BPM conditions using the Logic Pro software, which eliminates distortion in the recording and ensures the equivalence of the pieces, even when played at different tempos.

Dart-throwing program: following the three-day recruitment phase, the selected participants were placed in the Sports Science Laboratory where they completed the State-Anxiety scale (STAI) prior to the dart-throwing phases. The darts were thrown with the dominant hand, at a distance of 2.37 m from the front of the target, which was located 1.74 m from its center to the ground. The target was 42 cm in diameter with concentric circles listed from 1 to 9, with a central point of a value of 10. First, they completed a warm-up phase consisting of two series of three consecutive throws. Subsequently, they completed Phase 1 and Phase 2 of the dart-throwing task, each of which consisted of four series with three throws. Both the warm-up and Phases 1 and 2 of the dart throwing were executed with headphones on and with music in the corresponding cases.

In Phase 2, with the aim of controlling for the possible effect of the expectations produced after completion of Phase 1, at the end of the first phase they were informed of the score obtained and asked: What score do you think you will get in Phase 2? (Minimum 0 and maximum 120) and, how sure are you of getting it? (0 = sure and 10 = completely sure).

For each participant, we recorded the total time (minutes) taken to execute the throws (Phase 1 + Phase 2). After completing the warm-up phase, the participant was given the darts, informed of the task to be performed, and asked to “Take a position, and if everything is correct and you are ready, we will begin Phase 1 of the throws”. They were also informed that the time taken to complete the throws was being measured. This measurement finished when the final dart of Phase 2 hit the target. If a dart fell to the ground, the throw was repeated; otherwise it was marked as a valid throw. In order to reduce the possibility of researcher bias [27], all collaborators involved in the experimental set-up and measurements were unaware of the purpose of the study or the groups to which the participants belonged. In addition, the research collaborators were unable to hear the music during the execution of the test.

Approval for this study was obtained from the Andalusian Ethics Committee of Biomedical Research (Evaluation Committee of Huelva. Date of approval: 27/06/2019; Act: 7/19). The procedures used in this work adhere to the tenets of the Declaration of Helsinki of 1975, revised in 2013.

### 2.5. Data Analysis

Descriptive univariate statistics were computed for each study variable. Baseline between-group and within-group changes from Phase 1 to Phase 2 were assessed using 3 *×* 2 mixed factorial ANOVAs with a between-group factor (control without music group; slow-paced music group, fast-paced music group) and a within-subject factor (Phase 1 and Phase 2). Statistical assumptions were checked—including normality and homoscedasticity—and all assumptions were reasonably met. The criterion for statistical significance was set at 0.05.

Using the G* Power 3.1 (2017) sample size calculator, for an alpha error of 0.05, an effect size of 0.5 and a statistical power of 0.8 for ANOVA tests (Fixed effects, main effects, and interactions) for three study groups, a group size of 64 was estimated to be necessary. The initial sample was larger than the necessary size (*n* = 77). The statistical analyses were conducted using IBM SPSS Statistics 20 software (SPSS Inc., Chicago, IL, 2017).

## 3. Results

### 3.1. Preliminary Analysis

Descriptive statistics regarding socio-demographic variables, and those that were measured prior to the task, are presented in Table 1. ANOVAs and Chi-square tests conducted to compare the three randomized groups confirmed that there were no significant group differences in terms of these variables.

All three groups were shown to be equivalent in terms of the various inclusion criteria. It was observed that when they reported on the “frequency with which they have played darts in the last 5 years”, no significant differences were found between them (*χ^2^_(2,56)_* = 2.781, *p* = 0.249), and when asked “how much did you like the music you have heard”, with 0 indicating “I like it, but very little”, and 10 indicating “I like it very much”, there were no significant differences between the three groups, as revealed by an ANOVA (GC: *M* = 7.00, *SD* = 0.907; GS: *M* = 7.58, *SD* = 1.261; GF: *M* = 7.32, *SD* = 1.057; with an *F _(2,55)_* = 1.310, *p* = 0.278).

The participants did not differ in terms of prior musical experience (*χ^2^_(2,56)_* = 0.995, *p* = 0.608), whilst there were also no differences with regard to musical training (*χ^2^_(4,56)_* = 4.607, *p* = 0.330), or in the years of musical training received (*F _(2,55)_* = 0.159, *p* = 0.853) between GC (*M* = 0.72, *SD* = 1.841), GS (*M* = 0.95, *SD* = 2.068), and GF (*M* = 0.63, *SD* = 1.342).

When measuring the manipulation of expectations after finishing the test by asking “what do you think was the objective of the activity you have just carried out?” it was found that 94.64% (*n* = 53) stated that they thought “it was a test to assess the accuracy of the throws when wearing headphones to eliminate ambient noise ”, 3.57% (*n* = 2) thought that “it was an exercise to measure how they executed the throws” and 1.79% (*n* = 1) responded with “I didn’t know.”

### 3.2. Between-Group and Within-Group Differences at Phase 1 and Phase 2

To explore the possible differences between the study groups in the different phases, a mixed factorial ANOVA was conducted. The results revealed that there were no differences between groups (*F*_(2.53)_ = 1.193; *p* = 0.311), nor between phases (*F*_(1.53)_ = 3.050; *p* = 0.087). Similarly, analysis of the scores obtained by all groups in the two dart-throwing phases (see Table 2) using the Greenhouse–Geisser test revealed that the scores of the three groups did not differ, obtaining a statistic of *F*_(2.53)_ = 0.077 (*p* = 0.926).

### 3.3. Expectations of Outcomes in Phase 2

Finally, an ANOVA was conducted on the scores obtained by the participants when asked about the expectations of their results in Phase 2, the perceived certainty of achieving said results, and the time taken to execute the dart-throwing task. The results are displayed in Table 3.

Table 3 clearly shows that there were no statistically significant differences between the GC, GS, and GR groups in terms of the expectations of results or in the certainty of achieving said results. However, differences were found between the two phases in terms of total execution time, with a large effect size (*ŋ^2^_p_* = 0.143). Post-hoc comparisons of these times using the Bonferroni test revealed that GC = GS (*p =* 0.094), GS > GF (*p =* 0.020), and GC = GF (*p =* 1). Thus, the key difference is that the slow-paced music group (GS) took more time to execute the task in comparison with the fast-paced music group (GF).

## 4. Discussion

The present study attempted to evaluate the influence of music on performance, in this case when it is presented during a dart-throwing task. On the basis of previous work in the literature, we expected to find poorer performance—in terms of dart-throwing scores—in the group that threw darts in the presence of music in comparison with a control group who performed the task in the absence of music. We also expected to find that the group that heard slow-tempo music would achieve better results than the group that listened to high-tempo music. Finally, we hypothesized that the high-tempo music group would take less time to execute the task than the no-music control group and the slow-tempo music group.

The three groups of our study (GC, GS, and GF) obtained similar scores on the dart-throwing task, which suggests that—regardless of levels of general self-efficacy, state-anxiety, and optimism —the presence of music during the execution of a precision task, such as dart throwing, does not serve as a distractor [15,16] that hinders performance [5]. Therefore, at least in the present study, music did not exert a similar effect to that usually observed with background noise, that is, we did not find a decrement in performance on tasks that require considerable concentration and attention [15].

Our second prediction—that the slow-tempo music group would show superior performance on the dart-throwing task in comparison with the fast-tempo music group—was not supported by our findings, since the scores of the three groups, (GC, GS, and GF) did not differ in either Phase 1 or Phase 2. Moreover, our data are not in accord with the assertion of McPherson et al. [10] who stated that tempos slower than the physiological heart rate, compared with those above the heart rate, could improve parasympathetic activation and suppress sympathetic activation of the autonomic nervous system, which might be expected to produce superior performance in tasks that require attention, concentration, and precision. One feature of our procedure that could explain the absence of differences between the groups is that the BPM used in both groups is close to the physiological heart rate (60–105 BPM), which could facilitate similar levels of activation [10]. As mentioned earlier, some studies suggest that people have a preference for rapid tempos; however, Atan [30] states that if comparisons are carried out at very high tempos (200 BPM vs. 70 BPM) it will be difficult to observe differences in the performance of the groups. In this regard, when comparing the lower tempos (120–140 BPM) with a no-music condition, the former showed better performance on the tasks [31].

Finally, the third of our hypotheses was supported by the observation that GS, the group that listened to slow-tempo music, took longer to complete the dart-throwing task than participants in GF, the group that listened to fast-tempo music; however, this time did not differ from that shown by GC (without music).

These results partially support the notion of synchronization of motor behavior and the fact that below 60 BPM there does not have to be entrainment or physiological changes [2,9,16]. However, these data do not support the claim that motor entrainment is fostered by active interventions, as opposed to those in which there is an emphasis on passive listening without motor coordination [10], as in the case of our study. These results are consistent with the possibility that synchronizing the speed of movement with the tempo of music improves performance in gross motor tasks but not in those that require fine motor skills [10,11]. However, even though we used musical tempos that could have a relaxing effect on the participants [29], we have not found the improvements in performance reported in previous studies [14]. One possible reason for the discrepancy between our findings and the results reported by Kuan, Morris, Kueh, and Terry [14], is that in the latter study the music was presented prior to the dart-throwing task whereas in the present study it was presented during the execution of the task.

### Limitations and Future Research

The present study has several limitations. Previous studies have indicated the need to control for attentional focus, since differences have been found in the strategies used by throwers [32]. Dart throwing accuracy increases with an external focus [33], particularly when this focus is more distal (i.e., on the target) than proximal (i.e., on the trajectory of the dart) [34].

An additional limitation of this study could be the absence of male participants. As already mentioned, the evidence suggests that deliberate practice determines the differences in performance and skill levels, as opposed to the superiority of men over women [17]. For this reason, our sample consisted only of novice throwers, and there is also evidence to shows how professionals often present differences in terms of a variety of factors [35].

A limitation of the procedure used in our study, which should be considered in future research, is the absence of participants who knew the group to which they belonged (GF or GS), which would allow for measuring the role of the participants’ expectations regarding musical tempo and performance efficiency. It is also necessary to determine the BPM required in order for music to facilitate the execution of a task or, in contrast, to have a distracting effect on performance, as in the case of background noise [15,16].

## 5. Conclusions

In conclusion, our findings appear to support the notion that music heard during the execution of a sport in which closed motor skills are required (as in the case of dart throwing), does not act as a noise that interferes with or disrupts performance and efficiency. However, it has been observed that when participants listen to music at a tempo below the heart rate (which we considered to be a slow tempo in this study) they take longer to execute the task.

The results found in this work, where tempos very close to heart rate (60–105 BPM) have been used (which could be regarded as relaxing conditions), should be investigated in future studies using music played at a relatively faster tempo (>120 BPM). Similarly, studies of this sort should be replicated and extended to include other types of tasks that require closed motor skills.

## Figures and Tables

**Table 1:** Socio-demographic variables and optimism, state anxiety, and self-efficacy.

	TOTAL	GC	GS	GF	F_(2,55)_	*p*
	N = 56 M (SD)	N = 18 M (SD)	N = 19 M (SD)	N = 19 M (SD)		
Age	23.38 (6.77)	22.61 (2.66)	23.63 (8.23)	23.84 (8.06)	0.168	0.846
STAI-S	15.38 (8.52)	14.06 (6.98)	18.26 (10.91)	13.74 (6.57)	1.703	0.192
GSE	29.66 (3.46)	30.22 (3.77)	29.84 (3.04)	28.95 (3.61)	0.659	0.522
LOT-Optimism	10.77 (2.15)	10.28 (2.30)	10.74 (1.88)	11.26 (2.26)	0.974	0.384
LOT-Pessimism	7.71 (2.46)	7.28 (2.32)	7.42 (2.43)	8.42 (2.48)	1.249	0.295
LOT-Total Optimism	21.05 (3.89)	21.00 (4.07)	21.32 (3.82)	20.84 (3.98)	0.071	0.932

GC.—group control (without music), GS.—group with slow-paced music, GF.—group with fast-paced music, STAI-E.—State-Trait Anxiety Inventory (S.-State), GSE.—General Self-Efficacy Scale, LOT.—Life Orientation Test, M.—mean, SD.—standard deviation.

**Table 2:** Dart-throwing scores during Phase 1 and Phase 2 and interactions for all the outcomes.

	GC N = 18 M (SD)	GS N = 19 M (SD)	GF N = 19 M (SD)	F_(2,53)_	*p*
Score in Phase 1	47.83 (13.28)	42.89 (13.75)	46.58 (13.50)	0.077	0.926
Score in Phase 2	50.78 (11.89)	46.68 (14.62)	51.68 (11.99)		

GC.—group control (without music), GS.—group with slow-paced music, GF.—group with fast-paced music, M.—mean, SD.—standard deviation.

**Table 3:** Results of a between-group ANOVA to compare the variables of expected results, certainty of expected results, and time taken to execute the task.

	GC N = 18 M (SD)	GS N = 19 M (SD)	GF N = 19 M (SD)	F_(2,55)_	*p*
Expected results in Phase 2	45.56 (13.91)	45.74 (18.84)	47.26 (10.61)	0.075	0.928
Certainty of expected results in Phase 2	5.89 (1.68)	5.58 (1.92)	6.53 (1.07)	1.736	0.186
Total execution time (minutes)	2.68 (0.31)	3.00 (0.60)	2.60 (0.35)	4.426	0.017

GC.—group control (without music), GS.—group with slow-paced music, GF.—group with fast-paced music, M.—mean, SD.—standard deviation.
